# Contact toxicity of insecticides against rice weevil, *Sitophilus oryzae* L. and its effect on progeny production

**DOI:** 10.1038/s41598-024-80157-z

**Published:** 2024-11-18

**Authors:** C. N. Rajarushi, Suresh M. Nebapure, Ankur Biswas, S. Rajna, S. Subramanian

**Affiliations:** 1 Division of Entomology, ICAR-Indian Agricultural Research Institute, New Delhi, 110012 India; 2 ICAR-Indian Agricultural Statistics Research Institute, New Delhi, 110012 India

**Keywords:** *Sitophilus oryzae*, Contact insecticides, Lethal concentration, Exposure surface, Exposure time, Relative toxicity, Progeny production, Entomology, Agroecology, Toxicology

## Abstract

Post-harvest losses caused by insect pests, particularly the rice weevil, *Sitophilus oryzae*, pose a significant challenge in food storage facilities worldwide. To combat this pest, synthetic insecticides and fumigants are widely used. However, effective contact insecticides are scarce. Hence, the present study explored the alternatives by evaluating the contact toxicity of various insecticides against *S. oryzae* using glass, jute, and floor tiles as surface substrates and further examining the effect on progeny production of promising candidate insecticides. Among the insecticides tested, malathion exhibited the highest toxicity on glass and jute surfaces regardless of the exposure period. On the other hand, spinetoram proved to be the most effective on tile surface with an 8 h exposure period. Among the alternate insecticides (spinosad, spinetoram, chlorfenapyr and lambda-cyhalothrin), spinetoram was most effective with LC_50_ values of 155.8 and 116.9 mg/m² for 4 h and 8 h exposure, respectively, on tile surface; 204.6 and 129.0 mg/m² for 4 h and 8 h exposure, respectively, on glass surface; and 271.5 and 199.5 mg/m² for 4 h and 8 h exposure, respectively, on jute surface. Relative toxicity assessments revealed spinetoram to be 2.11 and 2.51 times more effective than deltamethrin on tile surface for 4 and 8 h of exposure, respectively whereas it was 1.14 times more effective than malathion on tile surface at 8 h exposure. Principal component analysis indicated a higher demand for insecticide doses closely associated with the structural properties of surfaces, particularly evident with jute surfaces. Furthermore, the effect on adult mortality and progeny production by malathion, spinetoram, and lambda-cyhalothrin revealed malathion as the most effective insecticide followed by spinetoram. Carboxylesterase, acetylcholinesterase, and Glutathione S-transferase (GST) activities were notably higher in deltamethrin-treated insects compared to other insecticides. The studies concluded that spinetoram can be considered an alternative to conventional insecticides for the management of *S. oryzae* under different storage conditions.

**Supplementary Information:**

The online version contains supplementary material available at 10.1038/s41598-024-80157-z.

## Introduction

The rice weevil, *Sitophilus oryzae* L. (Coleoptera: Dryophthoridae), is a devastating storage insect pest of cereal grains such as wheat, maize, barley, sorghum, and paddy rice^1,2^. It has a wide global range of occurrence, appearing in all warm and tropical sections of the planet^3^. Adults and larvae cause both quantitative (grain weight loss) and qualitative (nutritional and vitamin loss, as well as seed viability) loss leading to a significant drop in the commercial value of the food^4^. It has been reported to cause losses of 10–65% under moderate storage and up to 80% under prolonged storage conditions^4^. Besides direct damage, *S. oryzae* activity raises the temperature and relative humidity of stored grains promoting the establishment and development of fungal infections and causing grains to rot^5–7^ resulting in further losses.

Effective management of *S. oryzae* involves the use of fumigants such as phosphine and contact insecticides, used as grain protectants or as surface treatment in empty storage facilities. Residual sprays on storage facility surfaces ensure prolonged protection against reinfestation from hidden refugia and also offer a cost-effective solution, and leave minimal residues on food products^8,9^. In India, malathion (organophosphate) and deltamethrin (pyrethroid), contact insecticides, and phosphine, a fumigant are used to manage storage insect pests^10^. Indiscriminate use has led to the development of heritable resistance against phosphine in numerous stored product insect pests worldwide, including the rice weevil *S. oryzae*^11–16^ and long-term use of the same chemicals has resulted in reduced efficacy against the target pests in some places, possibly due to inappropriate application methods or insect resistance. This situation underscores the need to explore and identify alternate insecticides for potential rotation to manage resistance development effectively. These insecticides should exhibit contact toxicity, be cost-effective, easy to apply, and demonstrate prolonged residual action against a broad range of target species^17–20^.

Lambda-cyhalothrin, a pyrethroid insecticide with good contact and stomach action, is widely used against field crop pests and household pests^21^. Spinosad and spinetoram (spinosyn insecticides) are used worldwide in several crops^22,23^ and their potential in storage pest management through mixing and surface application has been reported^24–28^. Low persistency, relatively low mammalian toxicity, and good stability in storage grains^29^ make spinosyn insecticides a good candidate for storage pest management. Chlorfenapyr, a non-neurotoxic pyrrole insecticide that inhibits oxidative phosphorylation, has also been explored as a viable alternative insecticide for storage pest control^30–32^.

Several factors such as exposure surface, insecticide formulation, exposure period, variation in species susceptibility, and application rate determine the effectiveness of contact insecticides in storage facilities^33^. The range of surface substrates in storage facilities can be classified as non-porous surfaces viz., concrete, ceramic tile, galvanized steel, and porous surfaces viz., plywood, and jute bags, which poses a challenge to pest control programs because of their influence on insecticide toxicity^34–36^. We also assessed the impact of the best three insecticides on progeny production by treating the jute bags with LC_99_ values obtained from the bioassays to know the effectiveness of these insecticides in controlling the next generations of insects as well.

Variation in the susceptibility of stored product insects is primarily attributed to the continuous use of the same insecticides. This persistent usage has resulted in reduced sensitivity levels, posing significant challenges for effective pest management. Metabolic resistance, particularly mediated by detoxifying enzymes, plays a crucial role in insect variation in susceptibility to pesticides. Enzymes such as esterases/carboxylesterases, glutathione-S-transferases, and cytochrome P450 monooxygenases are recognized as key players in the detoxification process^37–39^. Hence, the present study also focused on profiling the activity of detoxifying enzymes *vis-à-vis* insecticides.

The present study aimed to screen insecticides belonging to Pyrethroids [lambda-cyhalothrin 5% EC], Spinosyns [spinosad 45% SC and spinetoram 11.7% SC], and Pyrrole [chlorfenapyr 10% SC] for their use as contact insecticide(s) on glass, jute, and tile surfaces against rice weevil, *S.oryzae* and to investigate their effect on progeny production. The outcome of this research will help to identify effective alternatives such as contact insecticides for use in bulk grain facilities.

## Materials and methods

### Insects

The freshly emerged adults of rice weevil, *S. oryzae* were obtained from colonies maintained in the Storage laboratory, Division of Entomology, ICAR-Indian Agricultural Research Institute (IARI), New Delhi (28.6377° N, 77.1571° E). The population was mass multiplied by rearing on whole wheat mixed with 5% Brewer’s yeast (wt: wt) at 26 °C ± 1 and 65 ± 5% relative humidity (RH) with 12:12 (L:D) photoperiod. For bioassays, adults of both sexes (mixed culture) of 2-week post-emergence were used.

### Insecticides

Six commercially available formulations of insecticides were used in the study namely, malathion 50% EC, deltamethrin 2.5% WP, spinosad 45% SC, spinetoram 11.7% SC, chlorfenapyr 10% SC and lambda-cyhalothrin 5% EC (Table 1). These insecticides were selected, based on current recommended practices in India and some possible alternative insecticides which have the potential to manage storage pests through contact toxicity as evident from the literature.

**Table 1:** Insecticides used for evaluation of contact toxicity against Rice Weevil, *Sitophilus oryzae* adults.

Insecticide name	Commercial name	Active ingredient (a.i. %)	IRAC main/sub-group	Mode of action
Malathion	Suthion	50EC	Organophosphates (1B)	Acetylcholinesterase (AChE) inhibitors
Deltamethrin	Shri-o-Thrin	2.5WP	Pyrethroids (3 A)	Sodium channel modulator
Lambda-cyhalothrin	Santri	5EC	Pyrethroids (3 A)	Sodium channel modulator
Spinosad	Tracer	45SC	Spinosyns (5)	Nicotinic acetylcholine receptor (nAChR) allosteric modulator
Spinetoram	Delegate	11.7SC	Spinosyns (5)	Nicotinic acetylcholine receptor (nAChR) allosteric modulator
Chlorfenapyr	Intrepid	10SC	Pyrroles (13)	Uncouplers of oxidative phosphorylation *via* disruption of proton gradient

The insecticides were grouped based on mode of action (IRAC, 2023); https://irac-online.org/documents/moa-brochure/ [Accessed on 5th Feb 2024].

### Surface substrates

Three different surfaces were screened to assess the efficacy of insecticides in this study. These surfaces were chosen as they are commonly found in bulk grain storage godowns and grain processing facilities. Surface substrates used in this study were prepared following Paudyal et al.^40^ for glass surface (Petri dishes made of Borosilicate glass, 90 mm in diameter, 15 mm high, and 63 cm^2^ surface), Swathikumari^41^ for jute cloth surface (circular disks 8.8 cm × 0.02 cm diameter), and Toews et al.^24^ for tile surface with some modifications. In each case the treated surface area was 60 cm^2^. Glass surface was used as a standard reference. Other surfaces like jute cloth, were used as grains are predominantly stored in gunny bags made of jute cloth in bulk grain storage godowns in Asian/South East Asian countries. The circular discs of the jute bag cloth material and Kota stone floor tiles were tightly held inside the glass Petri dishes by applying synthetic gums like flex Kwik (Pidilite Industries Ltd, Mumbai). The circular disks (8.8 cm × 1.2 cm diameter) floor tiles (made of *Kota stones*) procured from hardware stores were used for the evaluation of tile surfaces. The tile surfaces were cut into appropriate dimensions to fit well inside the Petri dishes. Molten paraffin wax was applied to seal the Petri dishes to prevent the escape of test insects.

### Bioassays

For all insecticides, 1% stock solution was prepared and subsequent doses were generated by serial dilution method with distilled water as the diluent. Preliminary range-finding tests were done to identify doses showing mortality within the 20–80% range for each insecticide. A range of concentrations was prepared for the test insecticides used for the bioassay on different surfaces (Supp. Table 1). Appropriate dilutions were made to obtain the required doses of insecticides. The insecticide solutions were uniformly sprayed on the test surfaces using a Potter Precision Laboratory Spray Tower (Burkhard Mfg. Co., Ltd.) at a pressure of 0.5 bar^42^. One milliliter (ml) of each dose was applied, with three replicates maintained for each dose. Surfaces sprayed with 1 ml of distilled water served as controls. The dosage in mg/m² was calculated based on the concentration of the spray solution. The test surfaces (glass and kota floor tile) were allowed to dry for 1 h (h) and the jute bag materials were dried overnight at 25 °C and 65 ± 5% RH^33^. Subsequently, 30 individuals were released onto each treated surface in the Petri dishes, and experiments were conducted for two exposure periods i.e., 4 and 8 h, after a specified period of exposure, the weevils were transferred to plastic culture vials (5 × 1.8 cm) (Axiva Sichem Pvt Ltd) containing fresh diet (whole wheat mixed with 5% yeast) and were placed in incubators at 25 °C and 65 ± 5% RH. The mortality of adults was recorded at 72 h after exposure for each treatment. The insects showing no movement or moribund were considered dead.

### Effect of selective insecticides on mortality and progeny production of*S. oryzae*

Based on LC_50_ values obtained in the contact toxicity bioassays, the three most effective insecticides viz., malathion, spinetoram, lambda-cyhalothrin were chosen for studies on determining the effect of insecticides on the progeny production of *S. oryzae*. Replicating the jute bags used for grain storage, mini jute bags of 1 kg capacity (18 cm×22 cm) were prepared and used for bioassays (Supp. Fig. 1). The jute bags were sprayed uniformly on the surface with LC_99_ dose of respective insecticide, separately and allowed to dry overnight. Each treatment was replicated thrice. The treated bags were filled with 1 kg of wheat grains The insecticide-treated bags with grains were placed in acrylic cages (45 cm × 45 cm × 60 cm) provided with adequate ventilation. The 200 individuals (mixed sex) of *S. oryzae* adult weevils were released inside the cage. All the cages were placed in insect growth chambers maintained at a light intensity of 15–20 lx, 28 ± 2 °C, 65 ± 5% RH, and a photoperiod (L: D) 12:12 h. The mortality of adult weevils was recorded on the surfaces of the bags/inside the cages wherein this experiment was conducted at 7, 14, and 21 days after exposure. After 21 d, all the dead and alive *S. oryzae* adults observed outside of the bags were removed and the bags were maintained in insect growth chambers for an additional period of 60 days to track the progeny production of the weevils considering the chance entry of weevils into the jute bags. The number of adults was counted at 60 d post-exposure in each treatment. The same set of experiments was conducted separately by using maize and rice grains as food substrates to estimate the effect of food substrate on progeny production. We ensured that the food grains used for this experiment were free from insect infestation. An untreated control was maintained under similar conditions for each set of treatments.

### Estimation of detoxification enzymes

The levels of detoxification enzymes were determined in *S. oryzae* after exposure to different insecticides. The adult weevils were exposed to LC_50_ values of respective insecticides obtained for 8 h exposure on glass surfaces and then transferred to plastic vials containing wheat grains as food substrate. After pre-determined time intervals of 12, 24, and 48 h, the survivors were used for estimation of detoxification enzyme activities. The whole-body tissues of *S. oryzae* were homogenized in 0.1 M sodium phosphate buffer with a motorized homogenizer at 4 °C conditions. The homogenates were centrifuged at 16,000 *g* for 20 min at 4 °C (Eppendorf, Centrifuge 58101 R, Germany). The supernatant was taken for biochemical analysis. The microplate spectrophotometer (Medispec, USA) reader was used for enzyme estimation at specified wavelengths for different enzymes as per the protocols mentioned hereunder. A blank was maintained for each study for comparison. Biochemical assays for carboxylesterase (CarE), glutathione-S-transferase (GST), and cytochrome P450 and acetylcholinesterase (AChE) assay were conducted following Kranthi et al.^43^, Habig et al.^44^. , Kranthi et al.^43^, and Ellman et al.^45^ with some modifications. The detailed protocols of detoxification enzyme assays are furnished in the Supplementary file.

### Statistical analysis

The mortality values were corrected as per Abbott’s formula^46^. The LC_50_ and LC_99_ values, 95% confidence limits, standard errors, the slopes of the regression lines, and χ2 significance tests, were estimated by probit analysis^47^ using PoloPlus 2.0 software (LeOra Software, California, United States). A three-way Analysis of Variance (ANOVA) was done to understand the main effect and interaction effect of surface types evaluated at two levels of exposure period (4 and 8 h). The treatment means were compared with Tukey’s Honest Significant Difference (HSD) test at α = 0.05 using SPSS 21.0. Percentage reduction in adult emergence of F1 progeny or inhibition rate (% IR) was calculated as % IR = (C_n_ –T_n_) 100/C_n_ (C_n_ is the number of newly emerged insects in the untreated bags and T_n_ is the number of insects in the insecticide-treated bags). One-way ANOVA with Tukey’s HSD test (*p* < 0.05) was used to compare the statistical significance of enzyme activities. Illustrations of relative toxicity were done using SPSS version 21.0. (SPSS Inc. Chicago, Illinois, USA). Pearson’s correlations analysis was used to correlate median lethal concentrations of different insectides using the “Metan” package. PCA biplots analysis used “FactoMineR” and “Factoextra” packages in R for dimension reduction. The relative toxicity of the test insecticide with reference to recommended insecticides i.e., malathion/deltamethrin was calculated using the following formula (using LC_50_ values).

$$\:\text{R}\text{e}\text{l}\text{a}\text{t}\text{i}\text{v}\text{e}\:\text{t}\text{o}\text{x}\text{i}\text{c}\text{i}\text{t}\text{y}=\frac{{\text{L}\text{C}}_{50}\:\text{o}\text{f}\:\text{R}\text{e}\text{c}\text{o}\text{m}\text{m}\text{e}\text{n}\text{d}\text{e}\text{d}\:\text{i}\text{n}\text{s}\text{e}\text{c}\text{t}\text{i}\text{c}\text{i}\text{d}\text{e}\text{s}\:}{{\text{L}\text{C}}_{50}\:\text{o}\text{f}\:\:\text{t}\text{e}\text{s}\text{t}\:\:\text{i}\text{n}\text{s}\text{e}\text{c}\text{t}\text{i}\text{c}\text{i}\text{d}\text{e}\text{s}}$$

## Results

### Toxicity of insecticides on different surfaces against adult*S. oryzae*

Range finding test was done to determine the range of doses for different insecticides (Supp. Table 1). The bioassay study was conducted to generate dose-response data for the *S. oryzae* against six insecticides for 4 h and 8 h exposure periods (Table 1). The results of the dose-response probit assay (Table 2) revealed that *S. oryzae* was showing varying levels of susceptibility to the test insecticides. The calculated χ2 value was lesser than the table value (χ2 = 12.59, df = 6 at *P* = 0.05) suggesting population homogeneity (Table 2) and henceforth, the probit model was found to be appropriate. The mortality response of *S.oryzae* varied significantly with the test surfaces and the insecticides. Malathion exhibited the highest toxicity on both glass and jute surfaces, with LC_50_ and LC_99_ values of 71.5 and 407.2 mg/m² (4 h) and 50.2 and 381.3 mg/m² (8 h) on glass, and 169.2 and 382.2 mg/m² (4 h) and 132.7 and 406.5 mg/m² (8 h) on jute. Deltamethrin displayed the lowest toxicity on both surfaces. Similarly on tile surface also, malathion exhibited the highest toxicity, with LC_50_ and LC_99_ values of 148.9 and 323.6 mg/m² (4 h) and spinetoram exhibited the highest toxicity, with LC_50_ and LC_99_ values of 116.9 and 329.9 mg/m² (8 h) (Table 2). Factorial analysis revealed that the mortality of *S. oryzae* was significantly affected by main effects viz., surface type, exposure period, and their associated interaction surface type × exposure period for all insecticides (Table 3).

**Table 2:** Comparative toxicity of insecticides against Rice Weevil, *Sitophilus oryzae* on different surfaces.

Surface		Insecticide	Exposure period	Slope	χ^2^ (df)	LC_50_ mg/m^2^ (95%CL)	LC_99_ mg/m^2^ (95%CL)	Heterogeneity	*P* value
Glass		Malathion	4 h	3.11 ± 0.27	1.05 (6)	71.5 (65.97–76.97)	407.2 (317.45–577.59)	0.17	0.991
			8 h	2.67 ± 0.24	0.26 (6)	50.2 (45.22–54.95)	381.3 (287.29- 569.79)	0.04	0.999
		Deltamethrin	4 h	12.86 ± 1.0	0.35 (6)	296.9 (291.21- 302.26)	446.8 (423.00–481.73)	0.05	0.969
			8 h	10.98 ± 1.0	1.57 (6)	281.2 (274.81–286.95)	450.4 (420.94–495.83)	0.26	0.873
		Spinosad	4 h	7.93 ± 0.67	0.72 (6)	230.8 (224.80–236.34)	395.7 (368.12–437.23)	0.12	0.989
			8 h	7.81 ± 0.66	1.01 (6)	182.5 (176.33- 188.23)	372.2 (337.66–426.20)	0.16	0.913
		Spinetoram	4 h	8.90 ± 0.72	0.91 (6)	204.6 (199.06–210.00)	373.7 (344.35–418.00)	0.15	0.955
			8 h	6.81 ± 0.54	1.37 (6)	129.0 (123.67–133.97)	294.9 (265.74–339.79)	0.22	0.796
		Chlorfenapyr	4 h	10.60 ± 0.89	0.52 (6)	234.6 (229.00–239.90)	392.4 (366.11–431.82)	0.08	0.949
			8 h	10.04 ± 0.75	0.72 (6)	180.6 (175.67–185.23)	312.0 (292.21–340.38)	0.12	0.983
		Lambda-cyhalothrin	4 h	7.13 ± 0.60	0.96 (6)	163.8 (157.62–169.45)	354.4 (319.77–408.47)	0.16	0.777
			8 h	7.57 ± 0.62	0.81 (6)	152.8 (147.29–158.15)	336.1 (302.86- 387.22)	0.13	0.842
Jute		Malathion	4 h	6.68 ± 0.58	0.80 (6)	169.2 (162.92–175.19)	382.2 (340.62–449.25)	0.13	0.999
			8 h	5.09 ± 0.50	1.06 (6)	132.7 (125.65–139.15)	406.5 (341.76–525.13)	0.04	0.967
		Deltamethrin	4 h	18.24 ± 1.52	0.56 (6)	448.3 (442.13–454.29)	610.4 (584.81–647.53)	0.09	0.960
			8 h	15.63 ± 1.45	1.09 (6)	412.1 (405.44–418.10)	578.4 (550.49–620.80)	0.18	0.883
		Spinosad	4 h	13.54 ± 1.16	0.62 (6)	314.0 (307.82–319.71)	477.0 (450.30–516.90)	0.10	0.981
			8 h	11.71 ± 0.97	0.62 (6)	278.1 (271.97–283.75)	437.5 (411.98–475.38)	0.10	0.984
		Spinetoram	4 h	11.57 ± 1.01	0.69 (6)	271.5 (268.25–278.64)	437.5 (398.04–456.80)	0.11	0.992
			8 h	8.70 ± 0.70	1.05 (6)	199.5 (193.67–204.90)	366.8 (338.63–409.18)	0.17	0.925
		Chlorfenapyr	4 h	14.71 ± 1.20	0.90 (6)	327.4 (321.37–333.25)	483.2 (460.99–521.94)	0.15	0.980
			8 h	15.18 ± 1.15	0.90 (6)	287.5 (282.18–292.28)	406.2 (389.86–429.09)	0.15	0.982
		Lambda-cyhalothrin	4 h	16.71 ± 1.37	1.25 (6)	379.1 (373.39–384.52)	523.9 (501.98–555.52)	0.21	0.970
			8 h	13.63 ± 1.22	1.07 (6)	331.0 (324.58–337.12)	506.3 (476.82 -550.57)	0.17	0.942
Floor tile		Malathion	4 h	7.10 ± 0.58	1.01 (6)	148.9 (143.3–154.16 )	323.6 (292.33- 371.91)	0.17	0.999
			8 h	5.21 ± 0.48	0.94 (6)	133.0 (126.52–139.11)	379.2 (325.60–471.24)	0.15	0.920
		Deltamethrin	4 h	14.16 ± 1.19	0.87 (6)	328.7 (322.44–334.56)	492.8 (466.61 -531.43)	0.14	0.825
			8 h	10.93 ± 1.03	0.62 (6)	293.7(286.98–299.66)	466.8 (436.86–513.15)	0.10	0.799
		Spinosad	4 h	9.92 ± 0.87	0.68 (6)	244.2 (237.65–250.0)	418.1 (388.45–463.50)	0.11	0.982
			8 h	8.38 ± 0.74	0.79 (6)	202.5 (196.21–208.50)	399.3 (362.37- 457.74 )	0.13	0.991
		Spinetoram	4 h	7.09 ± 0.60	1.07 (6)	155.8 (148.38–159.39)	348.0 (307.03–397.60)	0.17	0.951
			8 h	5.26 ± 0.45	0.60 (6)	116.9 (111.12–122.29 )	329.9 (286.01–402.66)	0.10	0.998
		Chlorfenapyr	4 h	10.84 ± 0.89	0.63 (6)	246.7 (240.81–252.21)	406.0 (380.19–444.35)	0.10	0.962
			8 h	9.22 ± 0.77	0.53 (6)	215.4 (209.57–220.91)	380.7 (352.84 − 422.63)	0.08	0.992
		Lambda-cyhalothrin	4 h	8.82 ± 0.75	0.44 (6)	216.4 (210.29–222.05)	392.1 (361.38 − 439.24)	0.07	0.994
			8 h	8.16 ± 0.69	0.93 (6)	180.9 (174.87–186.39)	354.3 (323.84–401.33)	0.15	0.987

Probit mortality and dose–response activity of *S. oryzae* on different surfaces. Adult weevils were exposed to insecticide-treated surfaces for 4 and 8 h, with motility recorded after 72 h. LC_50_, median lethal concentration (that would kill 50% of the treated population), similarly LC_99_ (that would kill 99% of the treated population). The fiducial limit is presented at the 95% confidence level; total 1080 numbers of insects were taken on each surface with seven doses of each insecticide; chi-square value (χ^2^) at 95% confidence level; *df* degrees of freedom (i.e. df = *n* − 2, where n is the number of concentrations administered for bioassay); p-value at 0.05 level of significance.

**Table 3:** ANOVA parameters for main effects and associated interactions of mortality values of Rice Weevil, *Sitophilus oryzae*.

Source	df	Malathion	Deltamethrin	Spinosad	Spinetoram	Chlorfenapyr	Lambda-cyhalothrin	Significance
		F value						*P* value
Surface type	2	1101.86	4271.57	5943.61	2793.18	812.45	2753.17	< 0.0001
Exposure period	1	213.72	475.19	1167.32	3139.76	353.01	191.61	< 0.0001
Surface type × exposure period	2	13.18	24.97	9.82	108.34	6.14	22.71	< 0.0001

The table presents the main effects viz., surface types (Glass, Jute, Tile) and exposure periods (4 h, 8 h), along with the associated interaction (surface type × exposure period) on mortality recorded at 72 h after treatments; Significance was determined using ANOVA and Tukey’s HSD test (*p* < 0.001); *df* degrees of freedom.

### Relative toxicity of insecticides on different surfaces against adult *S. oryzae*

**Fig. 1:**
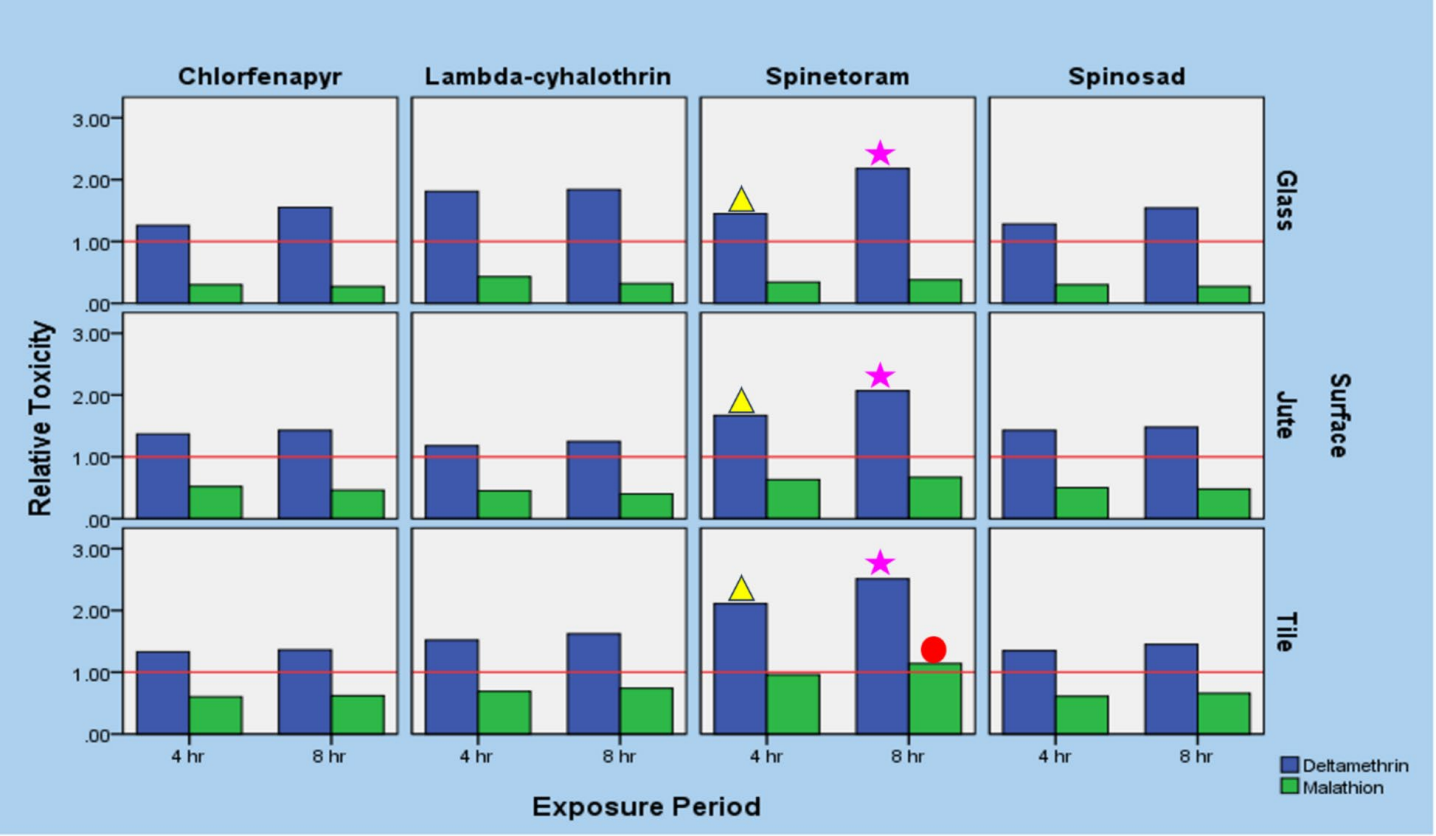
Relative toxicity of different insecticides against Rice weevil *Sitophilus oryzae* on different surfaces with reference to deltamethrin/malathion. Relative toxicity values calculated as the ratio of LC_50_ of deltamethrin / LC_50_ of each selected insecticide is denoted by blue bars; Relative toxicity calculated as the ratio of LC_50_ of malathion / LC_50_ of each selected insecticide is denoted by green bars. The horizontal red line at a value of one (or 1) serves as a reference, with values above indicating greater effectiveness than deltamethrin (blue) and malathion (green). The illustration was developed using SPSS version 21.0. (SPSS Inc. Chicago, Illinois, USA). (Yellow colour filled triangle) shows highly effective insecticide at 4 h exposure period on that particular surface among selected insecticide in relative to deltamethrin, and (Pink colour filled star) shows highly effective insecticide at 8 h exposure period on that particular surface among selected insecticide in relative to deltamethrin; (Red colour filled circle) shows highly effective insecticide at 8 h exposure period on that particular surface among selected insecticide in relative to malathion.

Relative toxicity of the test insecticides viz., spinosad, spinetoram, lambda-cyhalothrin, and chlorfenapyr was calculated in comparison to reference insecticides viz., malathion and deltamethrin (which are currently used in bulk storage godowns of food grains) (Fig. 1). Malathion is found to show better efficacy than all other test insecticides irrespective of test surfaces and exposure periods. It was also evident that spinetoram was most effective on tile surfaces with 0.96 and 1.14-fold toxicity relative to malathion, respectively for 4 and 8 h exposure periods (Fig. 1).

The relative toxicity values showed that all the test insecticides showed better efficacy than deltamethrin irrespective of surfaces and exposure periods (Fig. 1). On glass surface lambda-cyhalothrin was the most toxic with 1.81-fold better toxicity at 4 h exposure period, whereas at 8 h exposure period, spinetoram was the most toxic (2.18-folds). On the jute surface, spinetoram was the most effective showing 1.67 and 2.07-fold higher toxicity than deltamethrin respectively for 4 and 8 h exposure periods. Similarly, on tile surfaces too, spinetoram was found to be most effective with 2.11 and 2.51-fold higher toxicity than deltamethrin, respectively for 4 and 8 h exposure periods.

## Insecticide efficacy on various surfaces: PCA and correlation analysis

**Fig. 2:**
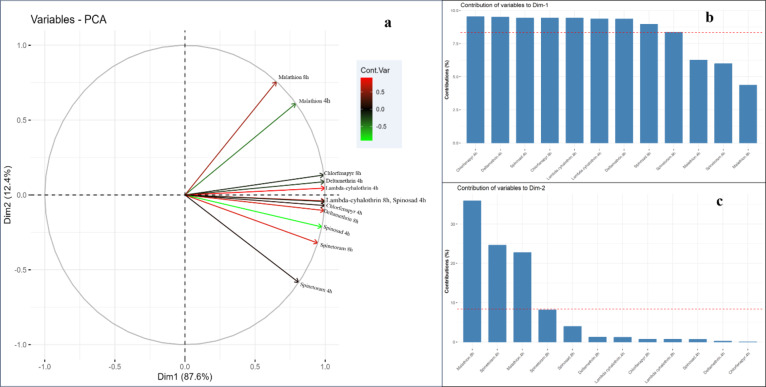
Biplot and contribution of insecticides to dimensions on different surfaces. (**a**) PCA biplot, Presentation of 12 variables viz., Malathion 4 h, Malathion 8 h, Deltamethrin 4 h, Deltamethrin 8 h, Spinosad 4 h, Spinosad 8 h, Spinetoram 4 h, Spinetoram 8 h, Chlorfenapyr 4 h, Chlorfenapyr 8 h, Lambda-cyhalothrin 4 h and Lambda-cyhalothrin 8 h; (**b**,**c**) contribution of 12 variables to dimensions one and two, respectively; PC1 and PC2 are represented on the horizontal and vertical axes, with a cumulative variance of 87.6% and 12.4%, respectively.

**Fig. 3:**
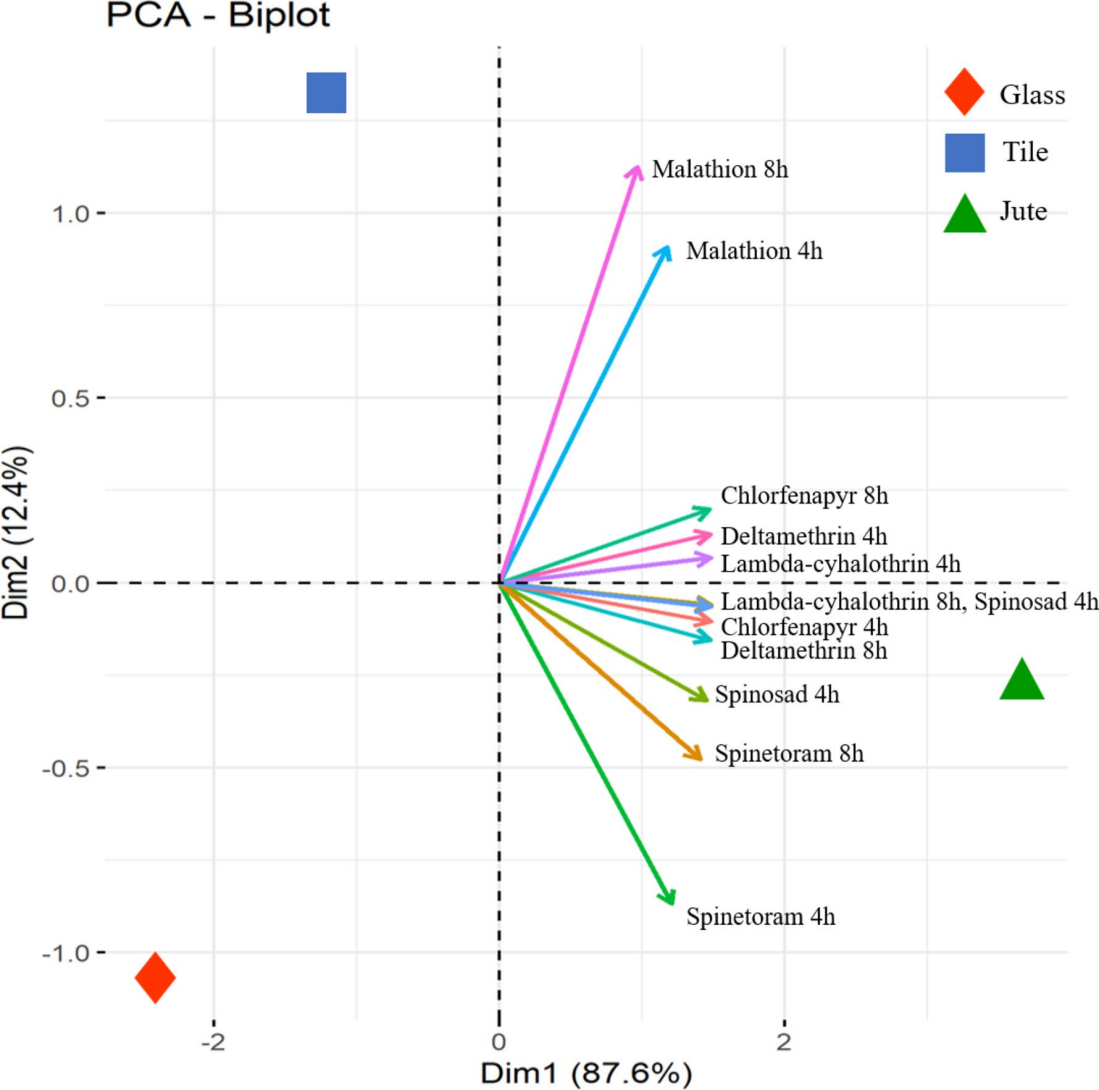
PCA analysis considering insecticides and surfaces (Glass, jute, and tile).Visualization of correlation showing the influence of surfaces on the toxicity of insecticides used in this study. The distance of the variables from the center indicates the magnitude of influence; PC1 and PC2 are represented on the horizontal and vertical axes, with a cumulative variance of 95% and 4.1%, respectively.

**Fig. 4:**
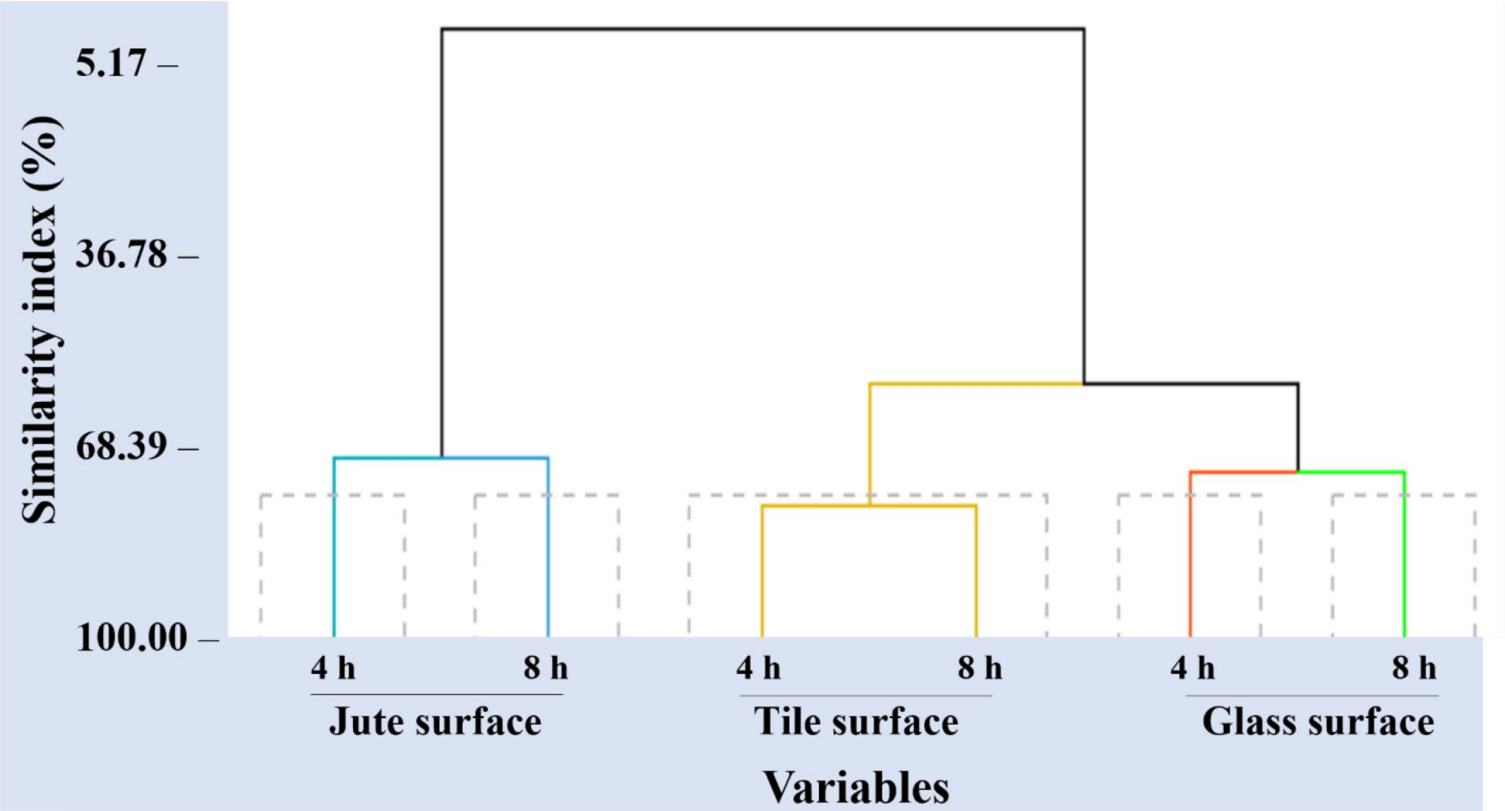
Cluster analysis shows the grouping of studied variables. Cluster analysis divided the whole group of variables into distinctly separated two major clades based on the similarity matrix: (Blue line) One was a cluster of jute surfaces, attributing variables encompassing six insecticides at two exposure periods and another (yellow line) was a cluster of tile surfaces and (black line) was a cluster of glass surfaces.

The Principal Component Analysis (PCA) assessed the cumulative efficacy of insecticides considering exposure periods (4 and 8 h) and test surfaces (jute, tile, and glass). The PCA identified two factors accounting for a cumulative variance of 100%. Dimension 1 (Dim 1) explained 87.6% of the variance, with significant contributors being chlorfenapyr 4 h and 8 h, deltamethrin 4 h and 8 h, lambda-cyhalothrin 4 h and 8 h, Spinosad 4 h and 8 h, and spinetoram 8 h. Dimension 2 (Dim 2) accounted for 12.4% of the variance, receiving contributions from spinetoram 4 h and malathion 4 h and 8 h (Fig. 2a-c). The eigenvalues for Dim 1 and 2 were 3.24 and 1.21, respectively (Table 4). The biplot showed a robust positive correlation among chlorfenapyr, deltamethrin, lambda-cyhalothrin, spinosad, and spinetoram in Dim 1, while Dim 2 highlighted malathion and spinetoram’s positive distribution, particularly on tile surfaces. Notably, the jute surface had a strong positive correlation with these insecticides, whereas the glass surface showed a negative correlation, implying higher efficacy at lower doses of glass (Fig. 3). Cluster analysis formed a distinct branch for the jute surface compared to other surfaces, reinforcing the PCA findings (Fig. 4).

**Table 4:** Eigenvalue, percentage of variance, and cumulative variance of PCA study.

Component	Eigenvalue	Variance (%)	Cumulative % of variance
Dim1	3.2426	87.6	87.6
Dim2	1.2188	12.4	100

This table provides the percentage variance, and cumulative variance for each component of PCA output; the variability in eigenvalues presents the reduction in dimension.

**Fig. 5:**
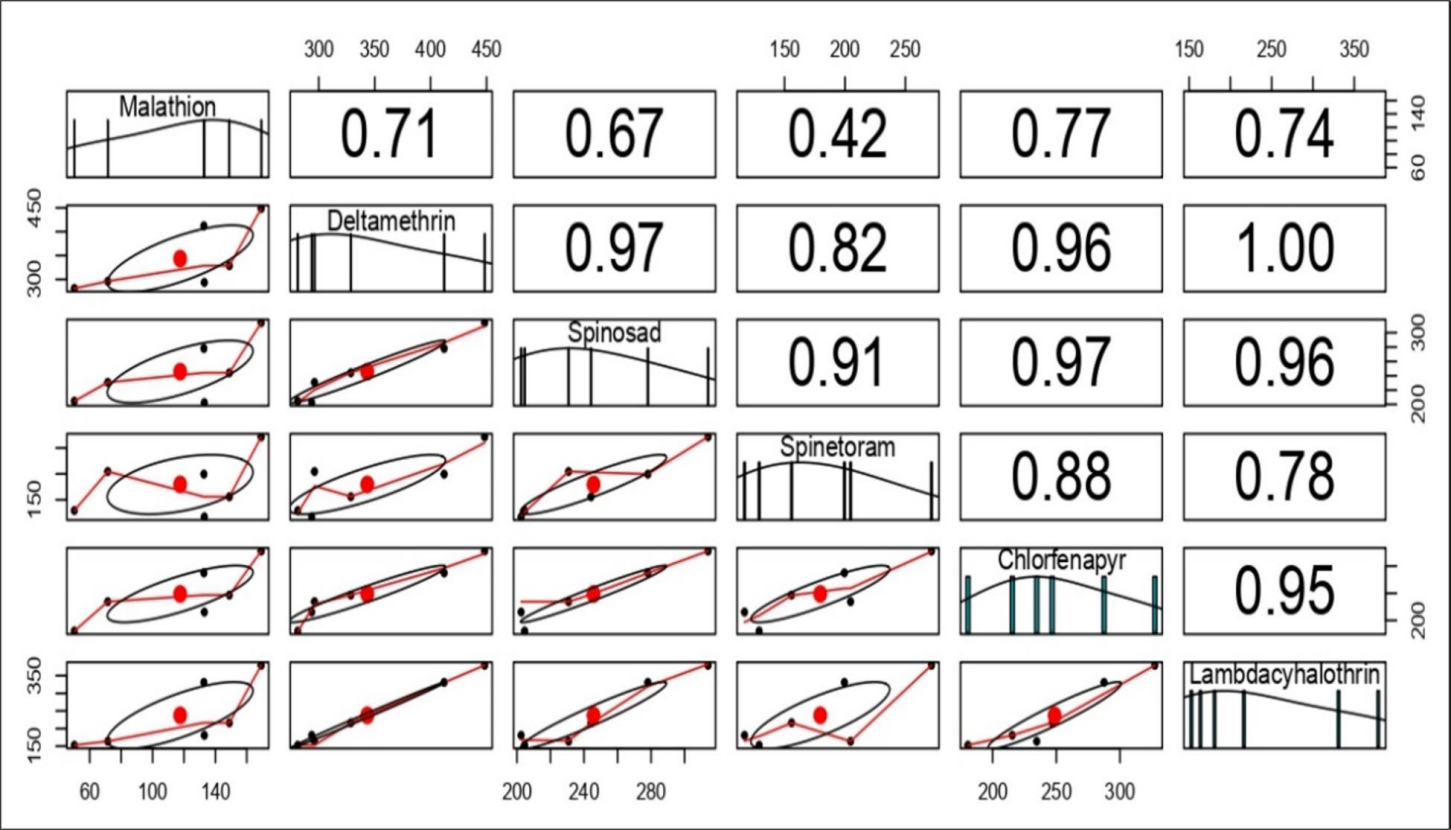
Pearson correlation coefficients for the median lethal concentrations (LC_50_) of various insecticides are presented in the upper half of the figure. These values indicate the strength and direction of the linear relationships between pairs of insecticides. A value close to 1 signifies a strong positive correlation, while a value near − 1 indicates a strong negative correlation; values around 0 suggest no significant correlation.

Pearson correlation analysis revealed robust and significant positive correlations at the 0.01 significance level (Fig. 5). A perfect positive correlation (*r* = 1.00) existed between deltamethrin and lambda-cyhalothrin, indicating precise alignment in their impact on LC_50_ mortality. Strong positive correlations were observed between deltamethrin-spinosad (*r* = 0.97), spinosad-chlorfenapyr (*r* = 0.97), deltamethrin-chlorfenapyr (*r* = 0.96), chlorfenapyr-lambda-cyhalothrin (*r* = 0.95), spinosad-spinetoram (*r* = 0.91), spinetoram-chlorfenapyr (*r* = 0.88), and deltamethrin-spinetoram (*r* = 0.82). Moderate correlations were found for spinetoram-lambda-cyhalothrin (*r* = 0.78), malathion-chlorfenapyr (*r* = 0.77), malathion-lambda-cyhalothrin (*r* = 0.74), malathion-deltamethrin (*r* = 0.71), and malathion-spinosad (*r* = 0.67). A weaker positive correlation was noted between malathion and spinetoram (*r* = 0.42).

### Effect of selective insecticides on mortality and progeny production of *S. oryzae*

**Fig. 6:**
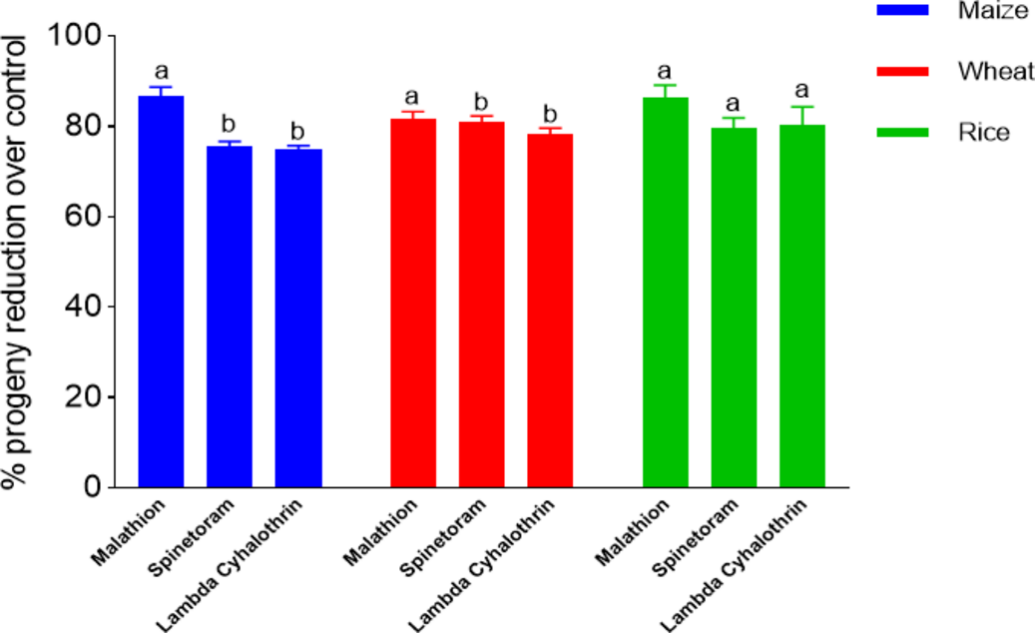
Effect of insecticides and food source on progeny production of Rice weevil, *Sitophilus oryzae*; Insecticides (malathion, spinetoram, and lambda-cyhalothrin) applied to jute bags containing three commodities (maize, wheat, and rice). Adult weevils were continuously exposed for 21 days, followed by the removal of live and dead parental adults. Progeny reduction was assessed at 60 days post-exposure. The same colour bars sharing a common letter indicate non-significant difference in progeny reduction over control (Tukey’s HSD test).

The study assessed the impact of highly effective insecticides, namely malathion, spinetoram, and lambda-cyhalothrin on the mortality of adult weevils and the progeny production of the weevils maintained on different food sources such as grains of maize, wheat, and rice. Significant differences in adult mortality were observed for these three insecticides. The progeny production was also found to be influenced by different food grains besides the toxic effect of insecticides. When maize was used as a food source, all insecticides caused significant adult mortality at 7, 14, and 21 days. Malathion caused the highest mortality rates of 67.8%, 76.6%, and 80% at 7 days (F = 198.30, *P* < 0.0001), 14 days (F = 129.70, *P* < 0.0001), and 21 days (F = 363.80, *P* < 0.0001), respectively. When wheat grains were used as a food source, all insecticides caused significant adult mortality at 7, 14, and 21 days, except for lambda-cyhalothrin and malathion at 21 days. Malathion resulted in the highest mortality rates of 60%, 71.1%, and 80% at 7 days (F = 47.07, *P* < 0.0001), 14 days (F = 76.29, *P* < 0.0001), and 21 days (F = 55.71, *P* < 0.0001), respectively. When rice grains were used as a food source, all insecticides caused significant adult mortality except for spinetoram and malathion at 14 days. Malathion induced the highest mortality rates of 68.9%, 74.4%, and 84.4% at 7 days (F = 69.79, *P* < 0.0001), 14 days (F = 101.1, *P* < 0.0001), and 21 days (F = 104.5, *P* < 0.0001), respectively. However, no significant difference was noted between spinetoram and lambda-cyhalothrin across the observation period in all the cases (Table 5). After 60 days, there was a reduction in progeny production on maize-filled jute bags: 86.80% for malathion, 75.73% for spinetoram, and 75.13% for lambda-cyhalothrin. Similar trends were observed in wheat and rice-filled jute bags (Fig. 6).

**Table 5:** Effect of insecticide-treated jute bags on mortality of Rice Weevil, *Sitophilus oryzae*.

Exposure	Insecticides	Commodity			F value	*P* value
		Maize	Wheat	Rice		
7 days	Malathion	67.8 ± 1.1^a^	60 ± 6.6^a^	68.9 ± 1.1^a^	2.77	0.140
	Spinetoram	53.3 ± 3.8^bA^	40 ± 1.9^bB^	50 ± 3.3^bA^	6.847	0.028
	Lambda-cyhalothrin	45.5 ± 1.1^b^	41.7 ± 1.6^b^	43.3 ± 5.7^b^	0.155	0.860
	Control	0.0 ± 0.0^c^	1.1 ± 1.1^c^	1.1 ± 1.1^c^	0.500	0.630
	F value	53.573	66.722	83.332	–	–
	*P* value	< 0.0001	< 0.0001	< 0.0001	–	–
14 days	Malathion	76.6 ± 5.1^a^	71.1 ± 1.1^a^	74.4 ± 2.9^a^	0.488	0.636
	Spinetoram	62.2 ± 2.9^b^	52.2 ± 4.8^b^	61.1 ± 4.0^ab^	1.872	0.233
	Lambda-cyhalothrin	61.1 ± 1.1^b^	52.2 ± 4.4^b^	58.8 ± 4.0^b^	3.701	0.090
	Control	0 ± 0^c^	2.2 ± 1.1^c^	1.1 ± 1.1^c^	1.500	0.296
	F value	137.852	75.225	93.996	–	–
	*P* value	< 0.0001	< 0.0001	< 0.0001	–	–
21 days	Malathion	60 ± 6.6^a^	80 ± 6.6^a^	84.4 ± 1.1^a^	1.906	0.229
	Spinetoram	40 ± 1.9^b^	58.8 ± 2.4^b^	65.5 ± 5.5^b^	4.088	0.076
	Lambda-cyhalothrin	41.7 ± 1.6^b^	67.7 ± 5.5^ab^	58.8 ± 4.0^b^	3.146	0.116
	Control	1.1 ± 1.1^c^	2.2 ± 1.1^c^	1.1 ± 1.1^c^	0.333	0.729
	F value	434.72	85.413	89.125	–	–
	*P* value	< 0.0001	< 0.0001	< 0.0001	–	–

Within each row, means followed by the same uppercase letter are not significantly different. Within each column, means followed by the same lowercase letter are not significantly different. Tukey’s HSD test at *P* = 0.05. Where no letters exist, no significant differences were recorded.

### Activity of detoxifying enzymes *vis-a-vis* insecticides in *S. oryzae*

**Fig. 7:**
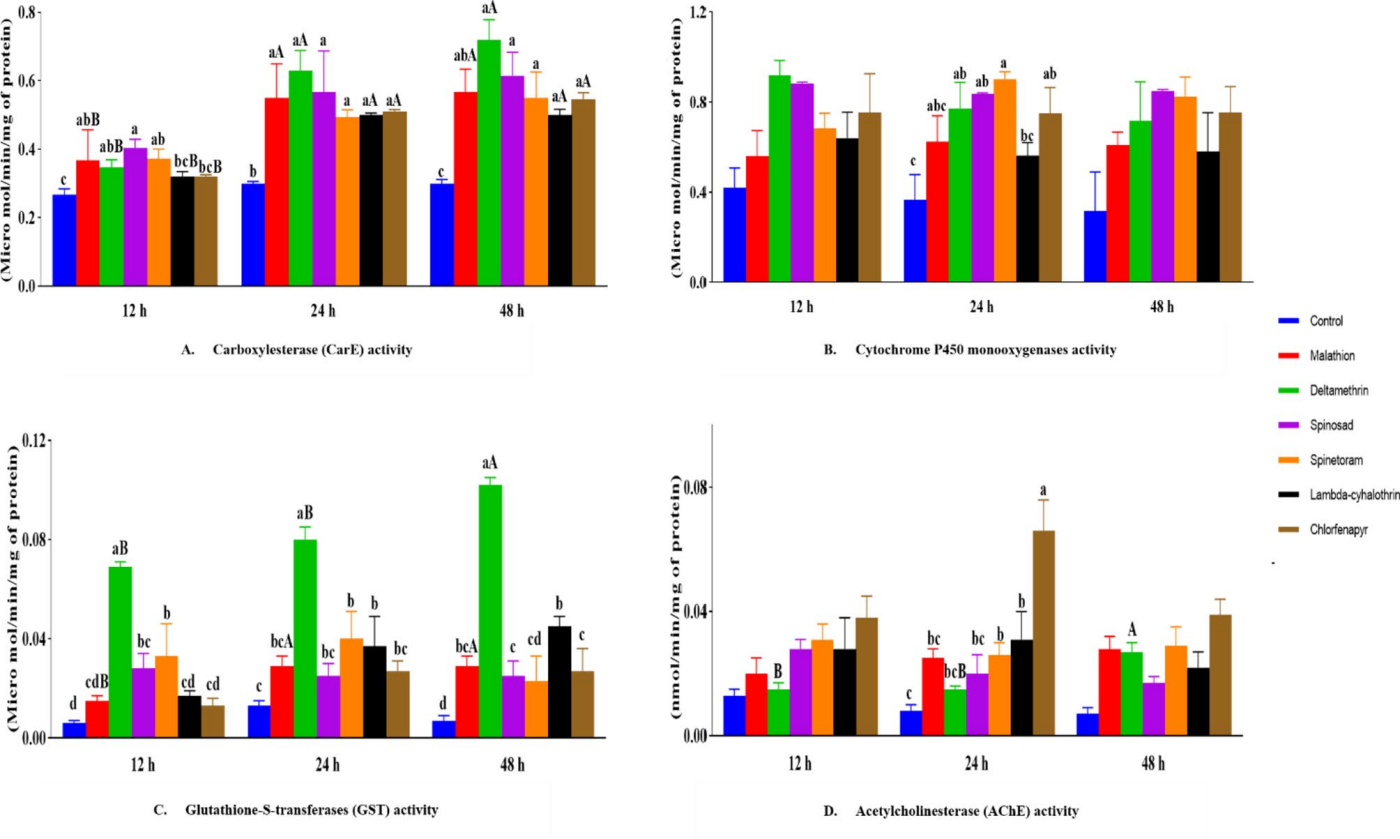
Detoxification enzyme activity expressed as micromole/min/mg of protein for Carboxyl esterase (**A**), Cytochrome P450 monooxygenase (**B**), Glutathione -S- Transferase (**C**), and as nmol/min/mg of protein for Acetylcholine esterase (**D**). Enzyme activity was recorded at 12, 24, and 48-h intervals. Statistically significant differences were determined by ANOVA and Tukey’s HSD test (*p* < 0.05), with shared uppercase letters indicating no significant differences within each insecticide across time intervals, and shared lowercase letters indicating no significant differences within each time interval across different insecticides. The absence of letters signifies no significant differences.

The activity of detoxifying enzymes such as carboxylesterase, cytochrome P450 monooxygenase, glutathione-S-transferase (GST), and acetylcholinesterase was estimated in adults of *S. oryzae* post-exposure to insecticides at LC_50_ doses after 12, 24 and 48 h, separately. Carboxylesterase and GST enzymes showed significant variations across all time intervals (Fig. 7). Carboxylesterase enzyme activity increased with an increase in exposure to food for all insecticides. At the 48 h exposure period, the highest carboxylesterase activity was recorded for deltamethrin (0.72 ± 0.058 µmol/min/mg of protein). Similarly, GST enzyme activity was highest in deltamethrin-treated insects across all exposure intervals, showing an increasing trend from 12 to 48 h (0.069 ± 0.002 to 0.102 ± 0.003 µmol/min/mg of protein). Significant variations in cytochrome P450 monooxygenase and acetylcholinesterase activities were evident only at the 24 h time interval (F = 3.804, *P* = 0.018; F = 10.284, *P* < 0.001). Cytochrome P450 monooxygenase activity displayed an increasing trend with exposure duration, with the highest activity observed for deltamethrin at 12 h, spinetoram at 24 h, and spinosad at 48 h. Acetylcholinesterase activity consistently peaked at 24 h for chlorfenapyr (0.066 ± 0.01 nmol/min/mg of protein).

## Discussion

In the present study, we delved into the realm of insecticides to unearth potential substitutes for malathion and deltamethrin, which have long been staples in combating storage insect pests across India. Furthermore, we investigated the impact of exposure duration and surface type on the mortality of *S. oryzae*. The effectiveness of insecticides is influenced by the surface treated^34,48^, the species of insect^24^, and the duration of exposure^49^. Our bioassay findings revealed that spinetoram and lambda-cyhalothrin exhibited higher mortalities and could be potential alternatives to malathion and deltamethrin which are being used in Indian bulk grain storage godowns over a long period of time. Several earlier studies have demonstrated the efficacy of spinetoram against stored product insect pests under short exposure intervals^50,51^. Athanassiou and Kavallieratos^52^ reported that spinetoram provided effective control of *Prostephanus truncatus* and *Rhyzopertha dominica*. Also, Toews et al.^24^ reported the efficacy of spinetoram on concrete, galvanized steel, unwaxed floor tile, and waxed floor tile surfaces against eight species viz., *Tribolium castaneum*, confused flour beetle, *T. confusum*, rusty grain beetle, *Cryptolestes ferrugineus*, merchant grain beetle, *Oryzaephilus mercator*, sawtoothed grain beetle, *O. surinamensis* and warehouse beetle, *Trogoderma variabile* at 0.05 and 0.1 mg ai/cm^2^. Complete control (100% mortality) of *S. oryzae* was observed on concrete and unwaxed floor tile for both dosages. Similar results on surfaces like plywood, ceramic tile, galvanized steel, and concrete surfaces against six stored product insect pests including *S. oryzae* reported by Vassilakos et al.^9^. wherein 100% mortality of *S. oryzae* adults was recorded on concrete surface treated with spinetoram at all dosage viz., 0.025, 0.05 and 0.1 mg/cm^2^. The efficacy of spinetoram on the concrete surface against *T. confusum* adults with 100% mortality of adults and larvae after 14 days was reported by Saglam et al.^53^. Gharib et al.^54^ reported that the residual toxicity of lambda-cyhalothrin on cement and polyethylene surfaces remained effective against *R. dominica* and *Callosobruchus maculatus* for up to six months maintaining 100% mortality at 0.479 g/m^2^. In a separate study, Khalequzzaman et al.^55^ demonstrated the toxic effects of lambda-cyhalothrin on *T. castaneum* on glass surfaces with LD_50_ value of 0.2416 µg/cm^2^. Additionally, Baliota et al.^56^ found lambda-cyhalothrin to be effective against *T. castaneum* and *O. surinamensis* on concrete.

Comparative efficacy of insecticides on different surfaces showed that the relative toxicity of all the tested insecticides was significantly higher on glass compared to floor tiles and jute surfaces. The higher efficacy on glass surface may be due to its non-porous nature avoiding migration and loss of insecticides. Besides, the frequent rolling behaviour of insects on glass surfaces could have resulted in higher contamination of cuticle with treated insecticide leading to higher mortality^57^. Relatively lower efficacy on other surfaces may be attributed to an increased rate of hydrolysis and degradation of the tested insecticides. Parkin^58^ reported that the hydrolysis of malathion on the tile surface led to rapid loss of toxicity. Our results also indicated that the toxicity of the tested insecticides on jute cloth surfaces was low. The reduced efficacy may be ascribed to the porous nature of the jute surface and absorption of chemicals resulting in lower mortality of the target insect. The jute surfaces necessitated larger deposits of insecticides as the molecules become firmly embedded in the jute fibers making them less accessible for insects through contact^58^. Our results also conform with the findings of Arthur^33,59^, Doganay et al.^60^, and Tsaganou et al.^61^ who have observed reduced toxicity of insecticides on linen fibers.

We observed that adult mortality of *S.oryzae* was increased as the exposure period was increased from 4 to 8 h. The increased exposure to the treated surface might have facilitated greater penetration of toxicant through insect cuticles. Similarly, Paudyal et al.^40^, Trdan et al.^62^, and Andric et al.^28^ also observed increased toxicity of the insecticides against *S. oryzae* and *S. zeamais* with the increase in exposure periods.

The PCA results indicate that the primary dimension (Dim 1) accounted for the majority of the variance (87.6%), with significant contributions from multiple insecticides over different exposure periods, emphasizing their overall efficacy. The second dimension (Dim 2), although accounting for a smaller portion of variance (12.4%), highlighted the distinct impact of spinetoram and malathion, particularly on tile surfaces. The robust positive correlation in Dim 1 among chlorfenapyr, deltamethrin, lambda-cyhalothrin, spinosad, and spinetoram suggests their similar modes of action and effectiveness across various surfaces. The negative correlation on glass surfaces implies that these insecticides are more effective at lower doses on glass compared to jute and tile surfaces, likely due to structural and textural differences. Cluster analysis further supported these conclusions, with the jute surface forming a distinct branch, indicating its unique interaction with the tested insecticides. These findings collectively emphasize the importance of selecting appropriate insecticides based on surface type and exposure duration to optimize pest control in storage environments. The Pearson correlation analysis reinforces these findings, showcasing strong alignments among several insecticides, particularly deltamethrin and lambda-cyhalothrin, which both belong to the sodium channel modulators group (IRAC classification 3a). This strong concordance underscores their similar effectiveness and potential for cross-resistance. In contrast, malathion displayed comparatively weaker correlations with the other insecticides, suggesting the potential replacement of malathion with newer chemistries for managing storage pests, including *S.oryzae*, in bulk grain storage.

The progeny production was suppressed to a greater extent in jute bags treated with the test insecticides than with untreated bags. Substantial reduction in progenies was observed with the treatments of malathion, spinetoram, and lambda-cyhalothrin. The food materials stored inside the jute bags also have an influence on progeny production by *S. oryzae* with wheat, maize, and rice. Athanassiou et al.^63^ reported that the survival and progeny production of *S. oryzae* was low in spinosad admixed wheat than in maize, rice, or barley. The insecticide treatments/exposures might have impacted the mating behaviour and offspring production of the stored product insect pests^64^.

The detoxification enzyme activity reported in this study reflects the physiological response of *S. oryzae* adults to different insecticides varying in their mode of action. Measuring enzyme activity at LC_50_, the concentration affecting 50% of the insects, ensures a consistent and reliable assessment of each insecticide’s inherent toxicity, providing accurate and comparable results^65^. Carboxylesterase, acetylcholinesterase, and Glutathione S-transferase (GST) activities were found to be particularly higher in deltamethrin treated insects when compared to other insecticides. However, there were fluctuations in detoxification enzyme activities at different time interval post-exposure to insecticides. Our results are in agreement with the finding of Mohan et al.^66^ and Zhang et al.^67^ who demonstrated the role of carboxylesterase in the detoxification of cypermethrin and deltamethrin in housefly, *Musca domestica* and cotton bollworms, *Helicoverpa armigera*. Earlier studies by Nazar et al.^68^ found increased AChE activity in chlorfenapyr-resistant *Phenacoccus solenopsis*. Kinareikina and Silivanova^69^ observed a 43.3% decrease in Vmax and a 1.88-fold decrease in Km in chlorfenapyr-resistant *M. domestica* females.

## Conclusion

Grain storage in India, with a capacity of 862.45 lakh metric tons, confronts severe challenges such as annual losses of 14 million tons worth Rs. 7,000 crore, primarily attributed to insect pests causing Rs. 1,300 crore in damages^70^, threatening food security. Traditional insecticides, once relied upon for their affordability and accessibility, are losing effectiveness due to resistance and safety concerns. Hence, there is a pressing need to adopt newer chemistries for more effective pest management while ensuring the safety of stored grains. Our experiment illustrated that spinetoram can be very effective as a contact insecticide against *S. oryzae* on common storage surfaces such as jute and tile compared to other selected alternatives. Nevertheless, additional experimentation is required to assess the spinetoram efficacy against other storage pests along with its persistence on treated surfaces in grain storage facilities. Our study offers valuable comparative data of spinetoram and other insecticides which can be a guideline for further research.

## Electronic supplementary material

Below is the link to the electronic supplementary material.

Supplementary Material 1

Supplementary Material 2

Supplementary Material 3

## Data Availability

The dataset generated during the current study are available from the corresponding author on reasonable request. All data generated during this study are included in this article.
