# Supplementary material for: Contact toxicity of insecticides against rice weevil, Sitophilus oryzae L. and its effect on progeny production
Source: Sci Rep. 2024 Nov 18;14:28404. doi: 10.1038/s41598-024-80157-z (PMC11574040; doi:10.1038/s41598-024-80157-z)
Supplement: Supplementary file 1 — Supplementary Material 1 [file 41598_2024_80157_MOESM1_ESM.docx]

**Supplementary Table 1.** Range of doses of insecticides used for bioassays on different surfaces (mg/m^2^).

| Insecticides | **Concentration of insecticides (mg/m^2^ ) tested on different surfaces** | | | | | |
| --- | --- | --- | --- | --- | --- | --- |
|  | Glass | | Jute | | Floor tile | |
|  | 4 h | 8 h | 4 h | 8 h | 4 h | 8 h |
| Malathion (50% EC) | 40-150 | 28-130 | 130-240 | 100-200 | 180-290 | 96-200 |
| Deltamethrin (2.5% WP) | 260-360 | 250-354 | 400-520 | 380-490 | 290-400 | 260-360 |
| Spinosad (45% SC) | 195-300 | 140-250 | 280-388 | 240-360 | 210-325 | 160-275 |
| Spinetoram (11.7% SC) | 160-268 | 102-200 | 230-340 | 160-280 | 120-225 | 88-195 |
| Chlorfenapyr (10% SC) | 200-300 | 150-260 | 290-400 | 260-370 | 210-325 | 180-290 |
| Lambda-cyhalothrin (5% EC) | 130-240 | 115-225 | 340-450 | 290-400 | 180-290 | 150-260 |

The table summarizes the experimental setup, featuring range of concentrations for each insecticide separately, tested on different surfaces (Glass, Jute, and Tile) under 4-hour and 8-hour exposure periods. Surfaces sprayed with distilled water served as the control.

**Detailed protocols of detoxification enzyme assays:**

**Carboxylesterase (CarE) activity**: CarE activity was studied using 1 mM α-naphthyl acetate as substrate and 1.0 % Fast Blue RR salt as staining solution. Absorbance was recorded at 600 nm for 10 min at a 1-minute interval by Microplate reader. Estimation of enzyme activity was done using a standard curve of α naphthol. The activity was expressed as µmoles of α-naphthol formed /min/mg protein.

**GST assay**: The activity of GST was determined spectrophotometrically at 25°C following the principle of glutathione (GSH) conjugate with 1-chloro-2,4- dinitrobenzene (CDNB) at 340 nm using the extinction coefficient of 9.6 mM-1 cm-1 (Habig et al. 1974). Briefly, 100 µL substrate was added to the total reaction mixture containing 100 µL of reduced glutathione and 100 µL of supernatant. The enzyme activity was expressed as µmoles of CDNB conjugates/min/mg protein. **Cytochrome P450 monooxygenase assay**: Cytochrome P450 activity was quantified using p-nitroanisole as a substrate (Yu et al. 1992). 2 mM p-nitroanisole, 9.6 mM NADH, and enzyme extract were used and the absorbance was recorded at 405 nm at 30°C. The activity was expressed as µmoles of p-nitroanisole/min/mg protein.

**Acetylcholinesterase (AChE) assay**: Acetylcholinesterase activity was determined by Ellman et al. (1961). 75 mM acetylthiocholine iodide was used as substrate for the enzyme. The assessment of enzyme activity was done by recording change in absorbance per min for 4 min at 410 nm. The activity was expressed as nanomoles of free thiol formed /min/mg protein.

**Protein estimation**: Total protein concentration was determined using the Bradford method (Bradford 1976) using bovine serum albumin as standard, and the absorbance was recorded at 595 nm.
