# Supplementary figures and images for: Contact toxicity of insecticides against rice weevil, Sitophilus oryzae L. and its effect on progeny production
Source: Sci Rep. 2024 Nov 18;14:28404. doi: 10.1038/s41598-024-80157-z (PMC11574040; doi:10.1038/s41598-024-80157-z)

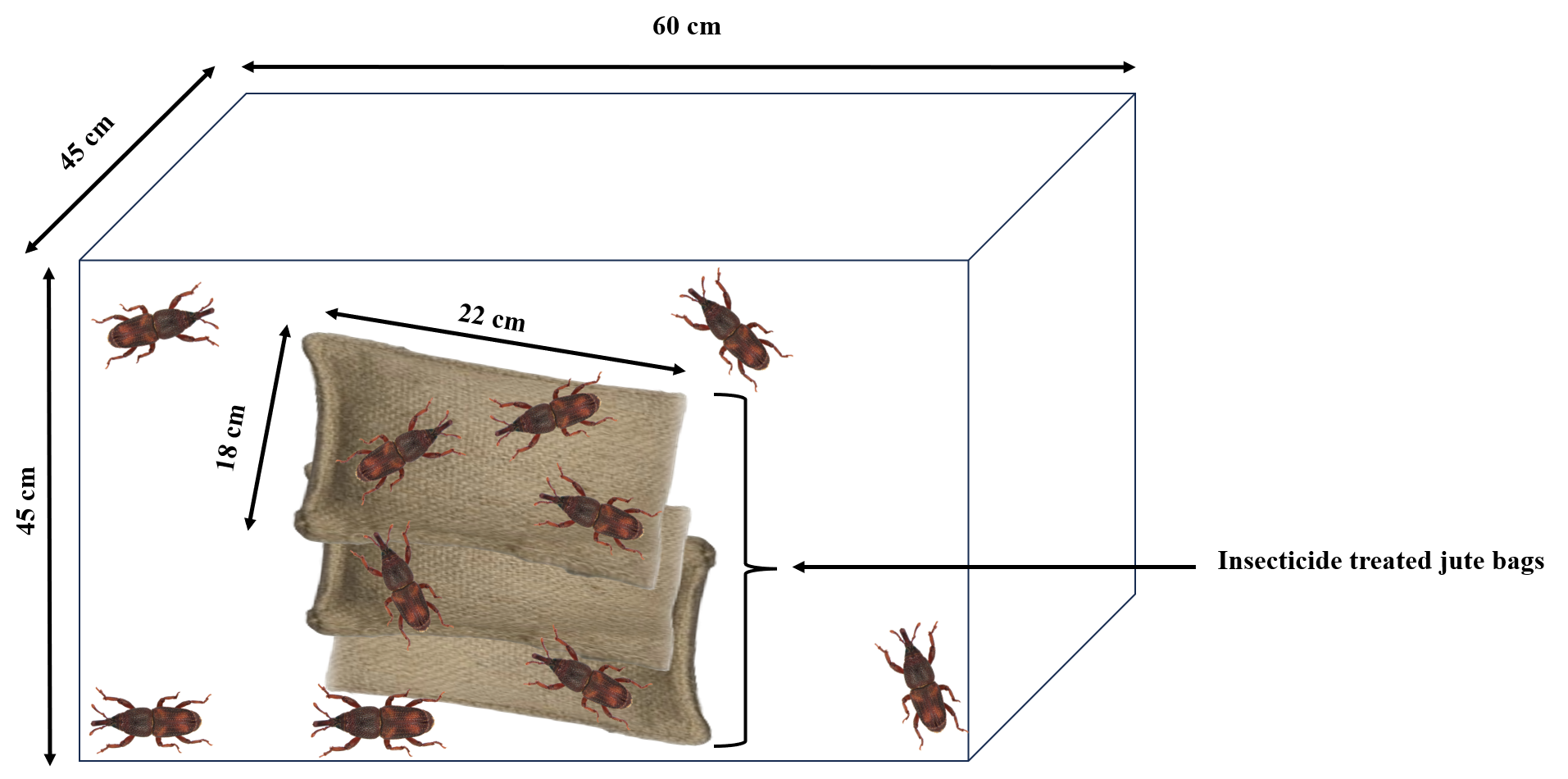

Supplement: Supplementary file 2 — Supplementary Material 2 [file 41598_2024_80157_MOESM2_ESM.tif]
