# Supplementary material for: Contact toxicity of insecticides against rice weevil, Sitophilus oryzae L. and its effect on progeny production
Source: Sci Rep. 2024 Nov 18;14:28404. doi: 10.1038/s41598-024-80157-z (PMC11574040; doi:10.1038/s41598-024-80157-z)
Supplement: Supplementary file 3 — Supplementary Material 3 [file 41598_2024_80157_MOESM3_ESM.docx]

**Supplementary figure 1**. Experimental set-up used for evaluation of insecticides effect on mortality and progeny production of *Sitophilus oryzae*. Jute bags were treated with various insecticides and evaluated for mortality of *S. oryzae* at 7-, 14-, and 21-days post-exposure. After the 21-day assessment period, the treated jute bags were transferred to polyacrylic cages and placed in insect growth chambers. The progeny production of *S. oryzae* was then monitored for a duration of 60 days.
